# Survival by histology among patients with bone and soft tissue sarcoma who undergo metastasectomy: protocol for a systematic review and meta-analysis

**DOI:** 10.1186/s13643-020-01445-z

**Published:** 2020-08-20

**Authors:** Ying Wang, Megan Delisle, Denise Smith, Amirrtha Srikanthan

**Affiliations:** 1 BC Cancer Vancouver, University of British Columbia, 600 West 10th Ave, Vancouver, V5Z 3E6 Canada; 2 General Surgery Residency Program Department, University of Manitoba, 347-825 Sherbrook Street, Winnipeg, MB R3T 2 N2 Canada; 3 Faculty of Health Sciences, Health Sciences Library, McMaster University, 1280 Main St W, Hamilton, Ontario L8S 4 L8 Canada; 4 The Ottawa Hospital Cancer Centre, University of Ottawa, 501 Smyth Road, Ottawa, Ontario K1H 8 L6 Canada

**Keywords:** Metastasectomy, Resection, Pediatric sarcoma, Soft tissue sarcoma, Bone sarcoma, Metastasis, Survival, Outcome

## Abstract

**Background:**

Metastasectomy is performed on a select cohort of patients with advanced and/or recurrent bone and soft tissue sarcomas because of the potential for long term relapse free and overall survival associated with the procedure. However, the evidence supporting metastasectomy is difficult to summarize without a systematic examination of existing literature. The objective of this systematic review will be to examine survival among both adults and children with advanced and recurrent bone and STS who undergo metastasectomy.

**Methods:**

We designed and registered a study protocol for a systematic review and meta-analysis. We will include data from survival studies (e.g., randomized trials, cohort studies, routine case registries, and case control) conducted in children and adults with advanced and recurrent bone and soft tissue sarcoma who undergo metastasectomy. The primary outcome will be overall survival. Secondary outcomes will be 30-day post-operative mortality, recurrence-free survival, time off systemic therapy, and patient-reported outcomes including quality of life end points where available. Literature searches will be performed in multiple electronic databases including Ovid MEDLINE ® (1946 to present), Ovid EMBASE (1974 to present), Web of Science, and Cochrane Library. Grey literature will be identified through searching references, conference abstracts, Papers First, and Google Scholar. Two investigators will independently screen all citations, full-text articles, and abstract data. Full-text articles selected for analysis will be assessed for quality and risk of bias. If feasible, we will conduct a random effects meta-analysis. Estimates will be stratified according to histology comparing survival based on organ of metastasectomy. Additional analysis will be conducted to explore the potential sources of heterogeneity according to various patient, disease, and treatment characteristics (e.g., metastasis status, age, disease burden, and concomitant interventions).

**Discussion:**

This systematic review and meta-analysis will identify, evaluate, and integrate data on survival of metastasectomy of bone and soft tissue sarcoma by organ of metastasis. Our findings may have implications for clinicians, patients, and their families when considering selection for resection of oligometastatic disease in de novo, or recurrent bone and soft tissue sarcoma. Implications for future research will be identified to improve the outcomes of these complex patients.

**Systematic review registration:**

PROSPERO CRD42019126906

## Background

Sarcomas are a relatively rare and highly heterogenous group of cancers originating from mesenchymal cells, haboring various histologies, and divergent natural history [1]. Bone and soft tissue sarcomas are two broad divisions within sarcoma. Approximately 20-50% of patients will develop metastasis from either bone or soft tissue sarcomas, presenting either as de novo or recurrent advanced disease [2–4]. Treatment of advanced, metastatic disease includes systemic therapy (such as chemotherapy, targeted therapy, and more recently immunotherapy), radiation therapy, ablation, and surgery [5–7]. Of the methods, surgical resection of metastasis (metastasectomy) is the accepted standard of care for a select cohort of sarcoma patients, as it is thought that, with careful clinical section, one can achieve long term relapse-free survival and potential cure [8].

The lung is the most common site of oligo-metastasis for bone and STS and has the most evidence supporting metastasectomy [9–11]. However, the evidence is currently limited to multiple retrospective cohort studies, with great emphasis on osteosarcoma as histology. Soft tissue sarcomas are distinct from osteosarcomas, with a wide variety of histologies further differing in biologic behavior within soft tissue sarcomas. For example, Van Geel et al. from the EORTC demonstrated a 5-year overall survival of 38% among patients who underwent complete pulmonary metastasectomy [12].

To date, two previous attempts have been made to systematically summarize survival statistics for pulmonary metastasectomy among patients with bone and soft tissue sarcoma, reporting 5-year overall survival of 20-34% for patients with bone sarcoma, and 13-25% for patients with soft tissue sarcoma [13]. These number demonstrate a wide range of survival and do not include survival outcomes for metastasectomy in extra-pulmonary sites. The current evidence is also limited in its assessment of the survival benefit of metastasectomy for extrapulmonary metastasis, such as hepatic or pancreatic metastasis [14, 15]. Thus, the evidence for pulmonary metastasis is extrapolated for patients with extrapulmonary metastasis. We hope to build up on current research and focus on reporting survival by individual histology comparing survival outcomes including patients with both pulmonary and extrapulmonary metastasis to facilitate accessible clinical information for individual patients.

This systematic review and meta-analysis will include patients with advanced bone or STS who undergo metastasectomy comparing overall survival among various clinical subgroups such as histology and location of metastasis so that patients with specific histologies are able to obtain a better estimate of survival. To our knowledge, a systematic review and meta-analysis has not been conducted on this topic.

## Methods

### Protocol and registration

This present protocol has been registered within the international prospective register of systematic reviews (PROSPERO) database (registration ID: CRD42019126906). The present study protocol is being reported in accordance with the reporting guidance provided in the Preferred Reporting Items for Systematic Review and Meta-Analysis Protocols (PRISMA-P) statement (see PRISMA-P checklist in Additional file 1) [16].

### Characteristics of participants/eligibility criteria

Only studies meeting the following criteria will be included:

- Patients: Articles with one or two treatment arms including patients with de novo or recurrent metastatic bone sarcoma including osteosarcoma, chondrosarcoma, Ewing sarcoma, rhabdomyosarcoma, or STS of various histologies (see Table 1 for details of specific STS histologies of interest). Patients of any age including children and adults will be included.
- Intervention: Articles including patients who undergo metastasectomy with the primary goal of increasing long-term survival.
- Comparators: Articles including patients who have metastatic bone or STS who do not undergo metastasectomy.
- Outcomes: Journal articles reporting on the survival outcome of the above-described patients, including reports of either median overall survival or survival follow-up of at least 1 year, whichever is the longer period to ensure an adequate amount of time has passed from time of metastasectomy.
- Other inclusion criteria: Peer-reviewed full text and original articles published in the English language.

**Table 1:** Eligibility criteria for inclusion into the systematic review

PICO	Inclusion criteria	Exclusion criteria
Population	- Either adults or children - Diagnosed with STS, including any of the following histologies: - Liposarcoma - Leiomyosarcoma - Undifferentiated pleomorphic sarcoma - Synovial sarcoma - Malignant peripheral nerve sheath tumor and triton tumor - Alveolar soft part sarcoma - Desmoid tumor - Solitary fibrous tumor/hemangiopericytoma - Fibrosarcoma or variants - Vascular sarcoma - Epithelioid sarcoma - Clear cell sarcoma - Desmoplastic small round cell tumor - Extraskeletal myxoid chondrosarcoma - Endometrial stromal sarcoma - Extraskeletal osteogenic sarcoma - Other STS not listed in the inclusion list - Diagnosis of bone sarcoma, including any of the following histologies: - Osteosarcoma - Chondrosarcoma - Ewing’s sarcoma - Rhabdomyosarcoma - Articles published up to March 31, 2019 - Articles published that incorporates either a comparison of, or independent listing of populations outlined within intervention and/or comparison group.	- Sarcomatoid epithelial tumors - Gastrointestinal stromal tumors
Intervention	- Studies including subjects who underwent pulmonary, hepatic, or other sites of metastasectomy for recurrent or de novo STS	Patients who underwent palliative surgery for symptomatic purposes without complete resection of metastasis (e.g., divert colostomy).
Outcomes	- Studies including survival measurement of either a comparison, or individual listing, of intervention and/or comparison group. - Secondary outcomes include 30-day post-operative mortality, recurrence free survival, time off of systemic therapy, and patient-reported outcomes including quality of life end points where available.	Studies that do not include mortality post metastasectomy as a study outcome

Articles will be excluded if:

- Focusing on evaluating the long-term outcomes of bone or STS undergoing curative-intent treatment without separately reporting survival outcomes for patients with resected recurrent/metastatic disease.
- Primary purpose of surgical resection of metastasis is not an attempt at the complete resection of metastasis to increase survival (e.g., surgeries for symptom palliation).
- Are abstracts, conference proceedings, editorials, letters, reviews, systematic reviews, and case studies containing less than five subjects.
- Full-text articles are not available after exhaustive searches to locate the texts.
- There were missing or insufficient data after a reasonable attempt at contacting primary authors.

### Information sources and search strategy

A structured search of major electronic databases will be performed in MEDLINE (Ovid), EMBASE (Ovid), Web of Science, and Cochrane Library. The secondary source of potentially relevant materials will be a search of grey or difficult to locate literature, including Google Scholar, Papers First, and conference abstracts from selected national and international symposia on oncology and surgery. For example, we will search for missed publications from presentation and abstracts in the last 3 years within the following: American Society of Clinical Oncology (ASCO) Annual Meeting, European Society of Medical Oncology (ESMO) Annual Meeting, Connective Tissue Oncology Society (CTOS), European Society of Surgical Oncology (ESSO) Annual Meeting, and Society of Surgical Oncology (SSO) Annual Meeting.

We will perform hand-searching of the reference lists of included studies, relevant reviews, clinical practice guidelines, or other relevant documents. Content experts and authors who are prolific in the field will be contacted if further expertise required outside of members of the group serving as content experts. The literature searches will be designed and conducted by the review team with the help of a health information specialist. A draft search strategy for MEDLINE is provided in Additional file 2. Search results will be uploaded to DistillerSR (DistillerSR, Evidence Partners, Ottawa, Canada).

### Screening and selection of studies

Following removal of duplicates, all articles identified from the literature search will be screened by two team members independently. First, the titles and abstracts of articles retrieved from the initial searches will be screened based on the eligibility criteria outlined above. Second, full-text articles will be assessed in detail and screened for eligibility. Third, references of key considered articles will be hand-searched to identify any relevant report missed in the search strategy. Any disagreements will be resolved by discussion to meet a consensus, if necessary. A flow chart showing details of studies included and excluded at each stage of the study selection process will be provided.

### Data collection/extraction

Two researchers will use a pre-set data extraction form to independently extract data on baseline patient, disease, and treatment characteristics, as well as data relevant to our primary and secondary outcomes. Journal information (name of journal, year of publication, authors, study design, country of origin), baseline patient characteristics (median age at diagnosis, median age at metastasectomy, gender, size of intervention, and control populations where applicable), disease characteristics (histology, site of primary sarcoma, stage and grade of disease at initial diagnosis, sites of metastasis, and disease-free interval), treatment characteristics (receipt of chemotherapy and/or radiation therapy for primary or metastatic disease, resection of primary disease, organ of metastasectomy, type of resection, number of metastasis resected, size of largest metastasis, operative approach, completeness of resection, additional metastasectomies, and other organ directed treatment(s) for recurrent disease), and outcome(s) post metastasectomy. The primary outcome will be overall survival. Secondary outcomes will be a 30-day post-operative mortality, recurrence-free survival, time off systemic therapy, and patient-reported outcomes including quality of life end points where available (Additional file 3). The data extraction form may be adapted during the extraction process in case new and relevant information arises. A pilot data extraction will be performed on five articles. Discrepancies between two evaluators in extracted data will be reviewed and resolved by a third evaluator. Reviewers will not be blinded to the authors, institutions, or journal of publication.

Where extra information is required, such as assessing detailed study data in published studies, a reasonable attempt will be made to contact the primary author of the publication via email. If no reply is received within 2 weeks from the time of initial contact, then no further attempts will be made to connect with study investigators, and involved studies will be excluded from the current systematic review.

### Risk of bias of individual studies

We expect that our studies will be observational studies (case control or cohort studies), thus risk of bias will be evaluated using the Newcastle-Ottawa Scale (NOS) adapted for cohort studies [17]. The NOS has a score range of 0-9 with articles scoring 7 or higher be considered as high quality. If there are eligible randomized controlled trials, risk of bias will be assessed using the revised Cochrane risk of bias tool for randomized trials RoB 2 [18]. Risk of bias will be assessed at the outcome level. Two researchers will conduct study quality assessments independently. Any disagreements will be resolved in consultation with a third team member or by group discussion.

### Data synthesis

Data presented in text and table form will be used to provide a qualitative description of included articles. The outcome study variables of interest are outlined above in the “Data collection/extraction” section and in Additional file 3.

Meta-analysis will be performed using a random-effects model to assess the pooled estimation of survival by the organ of metastasectomy, measured by rate ratio. Where available, hazard ratio will be estimated based on published Kaplan-Meier curves [19]. Statistical significance will be measured using 95% confidence interval (CI), and significance of the association set at *p* < 0.05. Heterogeneity between studies will be measured using the *I*-square statistic. In case of inadequate information, we will present data from individual studies without pooling for both primary and secondary outcomes.

### Subgroup analysis

Subgroup analysis will be conducted based on the number of studies available with appropriate outcomes and covariates. A priori subgroup and sensitivity analysis are proposed with clinical rational as follows:

- Age (adults vs. children): Our inclusion criteria include both children and adults to avoid excluding potentially useful information, this will be accounted for during data analysis, as pediatric sarcoma may receive different chemotherapy regimens than adults and have different prognosis given general lack of comorbidities among children. We plan to group all studies together initially by histology, then perform sensitivity and subgroup analysis by age group (e.g., age < 18 years versus 18 years or older), if possible.
- Year of publication (older vs. newer): Although the initial search is inclusive of all articles regardless of timing of publication, we plan on conducting stratified analysis on articles published from 1980 onwards to reflect survival in the more modern era of sarcoma therapies. This period was chosen to capture all relevant articles relating to the treatment of metastasis in bone and soft tissue sarcoma.
- Recurrent disease in the organ of metastasectomy only versus de novo metastatic disease will often entail different systemic therapies. For example, de novo metastatic disease will often receive both additional systemic chemotherapy and targeted treatment to the primary site of sarcoma. Thus, we will assess whether overall survival changes between these two categories of patients.
- For patients who undergo metastasectomy, we hypothesize that the organ of metastasis will also have an effect on our primary and secondary outcomes given the differing degree of difficulty in achieving a complete resection (e.g., metastasis in the lung versus brain). Given the predominance of literature supporting pulmonary metastasectomy, we plan on analyzing survival based on organ of metastasis: pulmonary vs central nervous system versus other organs undergoing metastasectomy.
- We hypothesize that disease burden will also have an effect on survival outcomes; thus, we would like to perform subgroup analysis based on a heavy versus light metastatic disease burden. The grouping of number of metastasis is to be specified within review.
- Although surgery is the mainstay of long-term survival for patients with metastatic sarcoma, other solid tumors such as colorectal cancer have demonstrated the improvement in survival when paired with peri-operative chemotherapy around the time of metastasectomy. If available, we would like to compare outcomes based on if patients received additional systemic or local therapies to surgery at the time of metastasectomy.

Data will be analyzed using STATA 14 (StataCorp. 2015. Stata Statistical Software: Release 14. College Station, TX: StataCorp LP). All results will be presented in accordance with the PRISMA guidelines.

### Meta-biases

Funnel plot symmetry and trim and fill method will be used to assess publication bias. This will help identify potential publication bias across studies and selective reporting within studies.

## Discussion

This described systematic review and meta-analysis will aim to provide a summation of the evidence of survival among advanced bone and STS patients who undergo metastasectomy and assess subgroups who may benefit more from the procedure. In an era with multiple local therapeutic modalities for treatment of metastatic disease, this article will provide a summation of work to spur on discussion for future prospective clinical trials.

Depending on the magnitude of the difference, our analysis may have implications for clinicians and patients when considering selection criteria for resection of oligometastatic disease in de novo, or recurrent bone and soft tissue sarcoma. These results may also help identify areas for further research to continue to improve the outcomes of these complex patients.

### Limitations

This systematic review is limited by the retrospective nature of anticipated articles. This will include greater potential for biases given the non-randomized nature of the studies. Access to large datasets such as individual participant data could further improve estimates to help shape how future trials might be undertaken. At the review level, we anticipate a large number of studies to qualify, and the need to break down this meta-analysis into several studies to allow for a clearer interpretation of data.

### Protocol amendments

Any major protocol amendments will be documented and submitted as a correction to the journal. Any minor protocol amendments will be referenced in the manuscript of the review.

## Supplementary information

**Additional file 1:.** PRISMA-P 2015 Checklist.

**Additional file 2:.** Search strategy for OVID Medline.

**Additional file 3:.** Key study variables and summary measures of interest to be extracted.

## Data Availability

The dataset generated and/or analyzed during the current study are available from the corresponding author on reasonable request.

## Abbreviations

ASCO: American Society of Clinical Oncology
CTOS: Connective Tissue Oncology Society
ESMO: European Society of Medical Oncology
ESSO: European Society of Surgical Oncology
PRISMA-P: Preferred Reporting Items for Systematic Review and Meta-Analysis Protocols
PROSPERO: International prospective register of systematic reviews
SSO: Society of Surgical Oncology
STS: Soft tissue sarcoma
