# Supplementary material for: Survival by histology among patients with bone and soft tissue sarcoma who undergo metastasectomy: protocol for a systematic review and meta-analysis
Source: Syst Rev. 2020 Aug 20;9:189. doi: 10.1186/s13643-020-01445-z (PMC7441630; doi:10.1186/s13643-020-01445-z)
Supplement: Supplementary file 2 — Additional file 2:. Search strategy for OVID Medline. [file 13643_2020_1445_MOESM2_ESM.docx]

Additional File 2. Search strategy for OVID Medline.

| 1 | exp Sarcoma/ |
| --- | --- |
| 2 | exp Nerve Sheath Neoplasms/ |
| 3 | exp Neoplasms, Vascular Tissue/ |
| 4 | exp Endometrial Stromal Tumors/ |
| 5 | sarcoma.ti,ab. |
| 6 | liposarcoma.ti,ab. |
| 7 | Leiomyosarcoma.ti,ab. |
| 8 | "malignant peripheral nerve sheath tum*".ti,ab. |
| 9 | desmoid.ti,ab. |
| 10 | hemangiopericytoma.ti,ab. |
| 11 | "solitary fibrous tum*".ti,ab. |
| 12 | fibrosarcoma.ti,ab. |
| 13 | "Desmoplastic small round cell tum*".ti,ab. |
| 14 | osteosarcoma.ti,ab. |
| 15 | Chondrosarcoma.ti,ab. |
| 16 | Rhabdomyosarcoma.ti,ab. |
| 17 | UPS.ti,ab. |
| 18 | triton tum*.ti,ab. |
| 19 | PNET.ti,ab. |
| 20 | "primitive neuroectodermal tum*".ti,ab. |
| 21 | "spindle cell tum*".ti,ab. |
| 22 | "soft tissue tum*".ti,ab. |
| 23 | "bone tum*".ti,ab. |
| 24 | Metastasectomy/ |
| 25 | Surgical Procedures, Operative/ |
| 26 | exp Neoplasm Metastasis/su [Surgery] |
| 27 | metastas*.ti,ab. |
| 28 | surg*.ti,ab. |
| 29 | 27 and 28 |
| 30 | resect*.ti,ab. |
| 31 | 27 and 30 |
| 32 | or/1-23 |
| 33 | 24 or 25 or 26 or 29 or 31 |
| 34 | 32 and 33 |
