# Supplementary material for: Survival by histology among patients with bone and soft tissue sarcoma who undergo metastasectomy: protocol for a systematic review and meta-analysis
Source: Syst Rev. 2020 Aug 20;9:189. doi: 10.1186/s13643-020-01445-z (PMC7441630; doi:10.1186/s13643-020-01445-z)
Supplement: Supplementary file 3 — Additional file 3:. Key study variables and summary measures of interest to be extracted. [file 13643_2020_1445_MOESM3_ESM.docx]

Additional File 3. Key study variables and summary measures of interest to be extracted.

| **Variable** | **Type** |
| --- | --- |
| **TRIAL INFORMATION** |  |
| Name of Journal | Free text |
| Year of publication | yyyy |
| First author | Free text |
| Study design | Free text |
| Country of study origin | Free text |
| Years of study | Numeric |
| **PATIENT CHARACTERISTICS** |  |
| Median age at diagnosis | Numeric |
| Median age at metastasectomy | Numeric |
| Sex  - Distribution of gender ratio of population |  |
| N  - Size of intervention population | Numeric |
| **DISEASE CHARACTERISTICS** |  |
| Histology | Categorical |
| Site of primary | Categorical:  - Extremity (details if extremity)  - Non-extremity (details if non-extremity)  - Visceral-gynecologic |
| Stage at diagnosis | I/II/III/IV/Unknown |
| Grade at diagnosis | 1/2/3/Unknown |
| Site(s) of metastasis with primary disease (if stage IV de novo) | Categorical: by organ of metastasis |
| Disease free interval if recurrent disease (months) | Numeric |
| **TREATMENT CHARACTERISTICS** |  |
| Neoadjuvant chemotherapy for primary disease | Y/N |
| Neoadjuvant radiation therapy for primary disease | Y/N |
| Resection of primary disease | Y/N |
| Primary sarcoma margin | Positive/Negative |
| Chemotherapy (adjuvant) | Y/N |
| Radiation (adjuvant) | Y/N |
| **METASTASECTOMY & PROGNOSTIC FACTORS** |  |
| “Neoadjuvant” chemotherapy for mets | Y/N |
| “Neoadjuvant” radiation for mets | Y/N |
| Organ of metastasectomy | Lung/liver/pancreas/other |
| Type of resection (%) | Text + % (e.g. wedge/segmentectomy/lobectomy/pneumonectomy + % of pts undergoing procedure) |
| Number of mets resected - average + range | Numeric +/- range |
| Size of largest mets (cm) | Numeric +/- range |
| Operative approach (%) | Minimally invasive vs open procedure (%) |
| Completeness of resection (%) | R0/R1/R2/Unknown |
| Additional metastasectomies - by organ (%) | Categorical - N (%) |
| Chemotherapy (palliative) | Y/N |
| Radiation (palliative) | Y/N |
| Other organ directed treatment for recurrent disease | Y/N |
| **OUTCOME POST METASTASECTOMY** |  |
| Post-operative complications | Categorical  - pneumonia/respiratory failure  - Atrial fibrillation  - Pleural effusion requiring chest tube  - Pneumothorax/persistent air leak  - Wound infection  - Empyema  - Colitis  - Wound dehiscence |
| Quality of Life Assessments pre and post metastasectomy (if included in study) | Free text |
| 30 day mortality (%) | Numeric |
| Recurrence free survival (months) | Numeric |
| Overall survival (months) | Numeric |
